# Effects of functional composition on plant competitors, stress‐tolerators, ruderals ecological strategies in forest communities across different climatic zones

**DOI:** 10.1002/ece3.11580

**Published:** 2024-09-03

**Authors:** Xin Han, Jie Yao, Ruixue Wang, Yue Xu, Jihong Huang, Yi Ding, Runguo Zang

**Affiliations:** ^1^ Forestry College of Shandong Agricultural University State Forestry and Grassland Administration Key Laboratory of Silviculture in Downstream Areas of the Yellow River Taian China; ^2^ Ecology and Nature Conservation Institute, Chinese Academy of Forestry Key Laboratory of Forest Ecology and Environment of National Forestry and Grassland Administration Beijing China; ^3^ Co‐Innovation Centre for Sustainable Forestry in Southern China Nanjing Forestry University Nanjing Jiangsu China; ^4^ Shandong Provincial Forestry Protection and Development Service Center Jinan China

**Keywords:** climatic zones, community functional structure, community‐weighted mean (CWM), ecological strategies, forest vegetation, Grime's CSR theory

## Abstract

Ecological strategies identified by plant functional traits are valuable descriptors for understanding species, populations, communities, and ecosystems in response to environmental conditions. Ecological strategies, in conjunction with the functional structure of plant communities, serve as crucial tools for investigating complex relationships among the environment, vegetation, and ecosystem functions. However, it remains unclear whether the functional structure (specifically, community‐weighted mean [CWM] traits) accurately reflects the optimal ecological strategies in forest communities. Here, we gathered seven functional traits for each species from four distinct forest vegetation types across four climatic zones, including leaf area (LA), specific leaf area (SLA), leaf dry matter content (LDMC), leaf phosphorus concentration (LPC), leaf nitrogen concentration (LNC), wood density (WD) and maximum plant height (H). We based on CSR (Competitors, Stress‐tolerators, Ruderals) theory and “StrateFy” ordination method utilizing LA, LDMC and SLA to position them within CSR triangle and categorize them into four ecological strategy groups: Competitive, Stress‐tolerant, Intermediate, and Ruderal ecological strategy groups (C‐group, S‐group, Int‐group, and R‐group). We then determined the proportion of species in each group. Subsequently, we calculated the CWM trait values for the remaining four functional traits: WD (CWM‐WD), LPC (CWM‐LPC), LNC (CWM‐LNC) and H (CWM‐H). Non‐metric multidimensional scaling and hierarchical partitioning revealed that CWM‐WD, CWM‐LPC, CWM‐LNC and CWM‐H significantly influenced the ecological strategies of forest communities. The synergistic interaction of CWM‐WD and CWM‐LPC had the most significant impact on ecological strategies within forest communities. Notably, CWM‐WD emerged as the most crucial single CWM trait for explaining variation in ecological strategies within forest communities. In conclusion, our study demonstrates that CWM traits effectively reflect optimal CSR ecological strategies in forest communities across different climatic zones, with CWM‐WD serving as a preferred indicator. This can improve our critical insights into key ecological processes in forest communities using trait‐based approach.

## Graphical abstract

Our study confirmed that community‐weighted mean (CWM) traits can well reflect optimal CSR (Competitors, Stress‐tolerators and Ruderals) ecological strategies in forest communities and CWM of wood density was a preferable indicator. These provided important implications for predicting community assembly responding to global change using trait‐based approach.

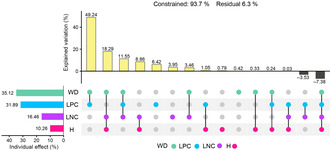

## 1 INTRODUCTION

In the context of increasing demands for global change and ecological conservation researches, there is a growing body of studies focusing on the impact of climate change on species distribution, community assembly and ecosystem function (Diaz & Cabido, 1997; Pierce et al., 2017). With the emergency of trait‐based ecology, our understanding of the mechanisms underlying community dynamics and changes in ecosystem function has significantly advanced (Chalmandrier et al., 2017, 2022; Lavorel, 2013). Ecological strategies, often referred to as plant functional types, can be defined as groups of species with similar combinations of functional traits (Guo et al., 2018; Han et al., 2021). These groups typically exhibit similar responses to environmental conditions and have analogous effects on key ecosystem processes (Diaz & Cabido, 1997; Grime, 1979). The functional structure of plant communities, which represents the distribution of trait values within these communities, significantly influences ecosystem processes and services (Borgy et al., 2017; Dias et al., 2013). In recent years, there has been a growing consensus that ecological strategies and the functional structure of plant communities can effectively be employed to explore the effects of environmental factors on vegetation and how vegetation, in turn, affects ecosystem properties (Borgy et al., 2017; Bruelheide et al., 2018). Consequently, it is reasonable to assume that investigating the relationships between ecological strategies and the functional structure of plant communities represents a promising avenue for understanding and predicting the variability in vegetation composition and ecosystem functioning within the context of global climate change.

The competitors, stress‐tolerators, ruderals (CSR) theory stands as one of the most comprehensive and influential theories regarding adaptive plant strategies currently (Wilson & Lee, 2000). The practical “StrateFy” tool as a crucial component of CSR theory is globally applicable to a wide range of life forms and has been subjected to global calibration and experimental validation (Li & Shipley, 2017; Pierce et al., 2017). It can efficiently classify a multitude of species into 19 distinct ecological strategy types, relying solely on three traits: leaf area (LA), leaf dry matter content (LDMC), and specific leaf area (SLA). Consequently, CSR theory in conjunction with the “StrateFy” tool provides ecologists with a common reference framework, facilitating quantitative comparisons of vegetation composition and community processes across highly contrasting habitats and various scales. (Grime & Pierce, 2012; Pierce et al., 2017). For instance, Rosenfield et al. (2019), drawing upon CSR theory, discovered that distinct forest types in transitional zones exhibited markedly different ecological strategies, even along a short latitudinal gradient. Zanzottera et al. (2021) demonstrated that diagnostic species in evergreen forest classes showed a convergence toward stress‐tolerant strategy, as opposed to deciduous forest classes that showed a tendency toward the competitive strategy. Wen et al. (2023), utilizing this framework, found that the ecological strategies of trees shifted from S/CS and CS strategies to CS/CSR strategies along the succession.

Examining consistent and predictable associations between plant ecological strategies and environmental conditions can enhance our understanding of species assembly within communities and shed light on how global changes and other anthropogenic factors impact ecosystem functioning (Bruelheide et al., 2018; Diaz et al., 1998). However, the 19 ecological strategy types based on CSR theory pose a challenge in matching them with specific environmental conditions when investigating general patterns between plant functioning and environmental conditions across diverse communities. To address this issue, in our recent research, we employed CSR triangle model to classify a diverse range of species into four ecological strategy groups to obtain ecological strategy spectrum for a given area or plant community (Han, Huang, et al., 2022). The ecological strategy spectrum offers a straightforward approach for exploring and understanding the biogeographical patterns of plant communities and the mechanisms behind their formation. For example, it has been demonstrated that this approach can be used to analyze the differences in composition of ecological strategy in typical forest vegetation across four climate zones and their relationships with abiotic and biotic factors (Han, Huang, et al., 2022; Han, Xu, et al., 2022). As a result, assessing how variation in functional structure of plant communities drives changes in species' ecological strategy through the lens of the ecological strategy spectrum represents a practical and feasible approach.

The community‐weighted mean (CWM) trait, calculated as the mean trait value for species within a community weighted by their abundances, stands as one of the most pivotal descriptors of community functional structure (Garnier et al., 2004; Violle et al., 2007). Utilizing CWM trait values offers deeper insights into species distributions, patterns of community diversity, and ecosystem properties in response to environmental conditions (Muscarella & Uriarte, 2016). Multiple studies have affirmed that considering species abundance strengthens the relationship between traits and the environment (Garnier et al., 2015). For instance, in comparison to the simple average traits of individual species, community‐level weighted averages of plant functional traits exhibit more robust correlations with insolation (Ackerly et al., 2002). Furthermore, community‐level traits more clearly reflect the response modes of plant traits to grazing compared to the species level (Cingolani et al., 2005). Additionally, CWMs of several fundamental vegetative traits have consistently shown associations with key ecosystem processes (e.g., Diaz et al., 2007; Garnier & Navas, 2012; Lavorel, 2013). For example, CWM of leaf nitrogen content and leaf tensile strength significantly affect litter decomposability, while CWM of leaf area and root length exhibit strong relationships with soil water content (Diaz et al., 2007).

The ‘CWM‐optimality’ hypothesis (Muscarella & Uriarte, 2016) posits that CWM trait values can mirror the locally ‘optimal’ trait strategies associated with species fitness, particularly when considering multiple traits. This suggests that community‐level traits and trade‐offs in multivariate traits closely intertwine with species' strong adaptation to specific environments. The ecological strategies rooted in trade‐offs among traits represent species' tactics for thriving and surviving in various habitats (Pierce et al., 2017). Optimal ecological strategies, therefore, can be viewed as the most effective adaptive ways that species develop for survival, growth and reproduction coping with their surroundings. Despite the frequent utilization of both ecological strategy and community functional structure in ecological issues, such as studying vegetation responses to disturbances (Barba‐Escoto et al., 2019; Ding et al., 2019) and the shifts of ecosystem function and services at a regional or global scale (Dias et al., 2013; Diaz & Cabido, 1997). However, the relationship between ecological strategy and community functional structure, and whether CWM traits can reflect optimal ecological strategies according to CSR theory, remain largely unexplored. Therefore, in this study, we established 200 20 m × 20 m forest dynamic plots (FDPs) in four forest types (50 in each forest type) across four climatic zones in China. A dataset that includes functional traits and species abundance of vascular plants was compiled. Subsequently, we based on CSR theory and the calculated CWM trait values to carry out our specific research. In the meantime, given plant height (H) is a key indicator for describing plant ecological strategies (Westoby, 1998) and relates to water availability and light competitive ability (Falster et al., 2017; Klein et al., 2015), which captures one of important aspects of plant function. And wood density has been identified as a crucial indicator of construction costs and structural integrity (Diaz et al., 2016), linking it positively to plant mechanical strength and resistance to biotic agents in woody species (Chave et al., 2009; Zanne et al., 2010). Our primary aim was to test the three following hypotheses:

1. CWM traits are directly associated with the ecological strategy spectra of forests. The distinctive functions and diverse responses to environmental conditions of individual functional traits result in varying effects of each CWM trait.
2. In a forest community, the CWM trait of maximum plant height is a more accurate indicator of species' optimal ecological strategy compared to the CWM traits of leaf (i.e., leaf phosphorus concentration and leaf nitrogen concentration), and stem (i.e., wood density).
3. Forest communities characterized by higher CWM traits of wood density tend to host a greater number of species employing an S‐selected strategy, while communities with elevated CWM traits of maximum plant height tend to accommodate more species employing a C‐selected strategy.

## 2 MATERIALS AND METHODS

### 2.1 Study site and tree census plots

The study was carried out in four typical forest vegetation types across four distinct climatic zones (tropical, subtropical, warm‐temperate, and cool‐temperate) from the south to the north in China. These typical vegetation types included: Tropical rainforest (TF) situated within Bawangling Nature Reserve in Hainan; Subtropical evergreen‐deciduous broadleaved mixed forest (SF) located in Mulingzi and Xingdoushan Nature Reserves in Hubei; Warm‐temperate coniferous‐broadleaved mixed forest (WF) found within Xiaolongshan Nature Reserve in Gansu; and Cold‐temperate coniferous forest (CF) situated in Kanasi Nature Reserve in Xinjiang. All typical forest vegetation types in our study are natural, undisturbed old‐growth forests, free from any human intervention.

We established a total of 200 0.04 ha (20 m × 20 m) forest dynamic plots (FDPs), with 50 FDPs in each forest vegetation type. For avoiding spatial pseudo‐replication, we randomly selected and ensured that the distance between each 20 m × 20 m plot within the same forest vegetation type exceeded 100 m. All FDPs were established and surveyed in accordance with the standardized protocols provided by the Center for Tropical Forest Science (http://www.ctfs.si.edu/). Within each plot, we meticulously identified all woody stems (excluding lianas) with a diameter at breast height (DBH, i.e., diameter at 1.3 m above the ground) ≥1 cm. We recorded their DBH, noted their precise coordinates, and estimated their height. We identified all species present in our study to species level and standardized their botanical nomenclature referring to the Flora of China (http://www.efloras.org). The identify of species in the filed investigation relied on our investigation team and the assistance of local botanists: the Xiusen Yang in Hainan, Yongmei Yi in Hubei Province, Anmin Li in Gansu, respectively. During the filed investigation of Xinjiang, the identifications of plant species depended on our investigation team. In this study, we mainly focus on woody species because they best represent the physiognomy and structure of forest communities they characterize.

### 2.2 Functional traits

We totally measured seven functional traits, including five leaf traits: leaf area (LA, mm^2^), specific leaf area (SLA, mm^2^ mg^−1^), leaf dry mass content (LDMC, %), Leaf nitrogen concentration (LNC, g kg^−1^) and leaf phosphorous concentration (LPC, g kg^−1^); one stem traits: wood density (WD, g cm^−3^); one whole‐plant traits: maximum plant height (H, m). For trait measurements of leaf and stem, we sampled evenly 10 individuals of each species across 50 FDPs in each forest vegetation type and then selected five mature healthy leaves and one 5 cm long branch with diameter between 1 and 2 cm from each of the five individual plant. For trait measurements of H, we selected evenly five tallest mature individuals of each species across 50 FDPs in each forest vegetation type and utilized clinometer to calculate their height. For species which were fewer than 10 and five individuals, we sampled all individuals for trait measurements. The collection of samples and measurement of functional traits were carried out at the peak of the growing season (between August to October from 2017 to 2019). Full details on trait measurements referred to the standard protocols (Pérez‐Harguindeguy et al., 2013).

Briefly, LA (mm^2^) is vital for intercepting light and the most common metric for size spectrum (Diaz et al., 2016). SLA (mm^2^ mg^−1^) is positively related to potential relative growth rate (RGR; Pérez‐Harguindeguy et al., 2013). LDMC (%) correlates negatively with potential RGR and positively with leaf lifespan (Pérez‐Harguindeguy et al., 2013). SLA and LDMC generally represent the opposite extremes of the economics spectrum (Pierce et al., 2017). LA, SLA and LDMC are used to identify the ecological strategies of species surviving in habitats (Pierce et al., 2013, 2017). LNC (g kg^−1^) and LPC (g kg^−1^) are tightly linked to plant growth and play crucial roles in ecosystem function and dynamics (Chen et al., 2013; Sterner & Elser, 2002). WD (g cm^−3^) is a core functional trait relating to the growth‐mortality trade‐off, which is important for the stability, defense, architecture, hydraulics, C gain and growth potential of plants (Pérez‐Harguindeguy et al., 2013). H (m) is associated with competitive ability, reproductive size and potential lifespan (Pérez‐Harguindeguy et al., 2013). We used the mean functional trait values of all individuals in each species to represent functional trait values per species.

### 2.3 Ecological strategy and ecological strategy spectra

**FIGURE 1:**
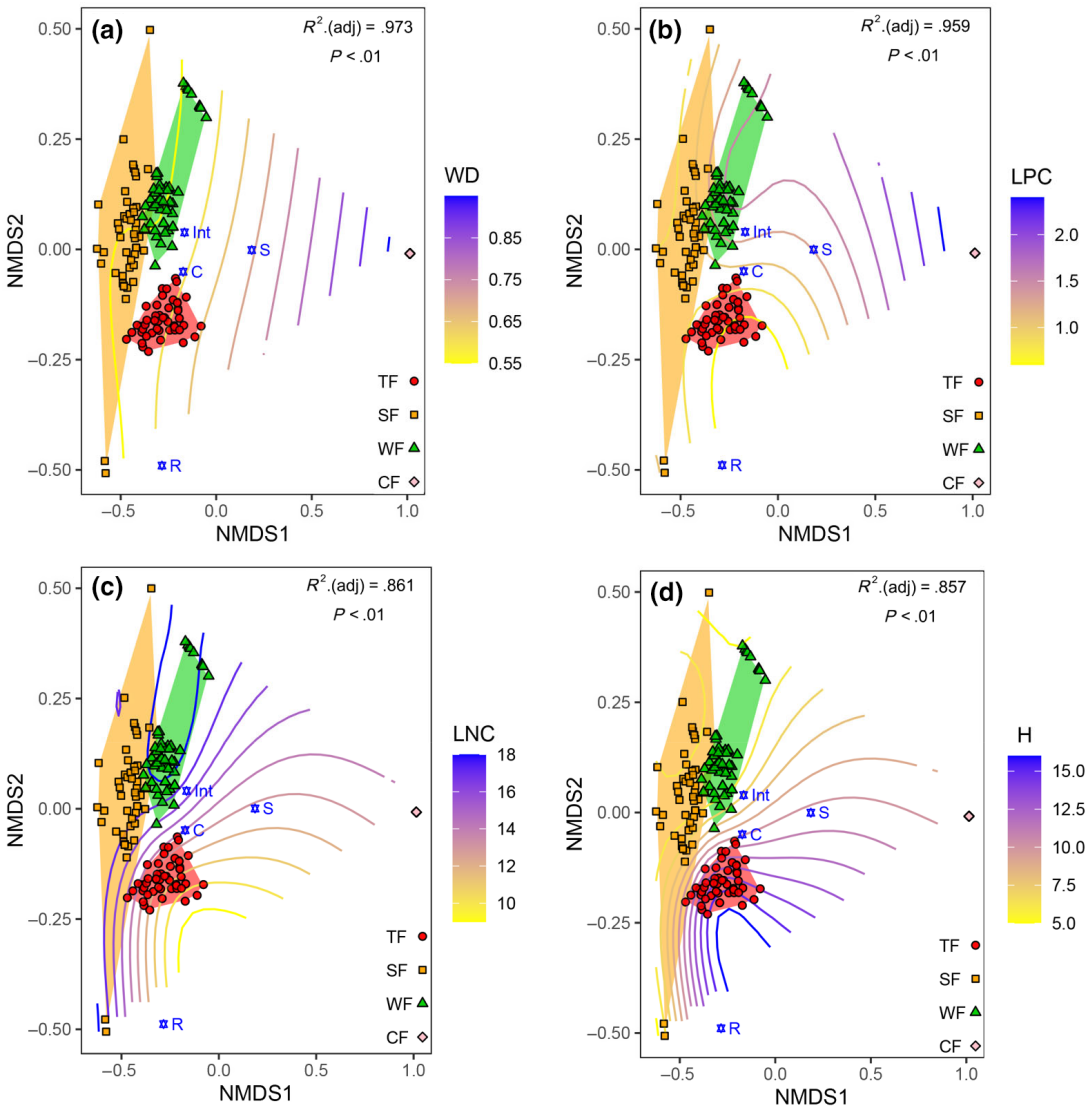
Non‐metric multidimensional scaling (NMDS) FDPs based on the ecological strategy spectra, with smooth response curves of the CWM traits overlain. (a) CWM trait of wood density (WD); (b) CWM trait of leaf phosphorus concentration (LPC); (c) CWM trait of leaf nitrogen concentration (LNC); (d) CWM trait of maximum plant height (H). TF, SF, WF and CF with different colors and shapes represented different forest vegetation types: TF, red, circles, tropical forest; SF, yellow, squares, subtropical forest; WF, green, triangles, warm‐temperate forest; CF, pink, diamonds, cold‐temperate forest. C, S, Int and R with blue indicated different ecological strategy groups: C, competitive ecological strategy group; S, stress‐tolerant ecological strategy group; Int, intermediate ecological strategy group; R, ruderals ecological strategy group. WD, LPC, LNC and H represented the different CWM traits: WD, wood density. The color gradient represented variation of CWM traits: blue represented increased CWM trait values and yellow represented decreased. *p* < .01 indicated a significant effect. All FDPs in CF (pink diamonds) almost had similar ecological strategy spectra, thus, the point in fact, was the overlap of 50 points.

We used “StrateFy” (Pierce et al., 2017) basing on the three leaf traits: LA, LDMC, SLA to identify the ecological strategy of each species and assign it in CSR triangle. And then, we connected the midpoints of three axes of the CSR triangle to divide the ternary plot into four parts obtaining four resultant strategy groups: competitive ecological strategy group (C‐group), stress‐tolerant ecological strategy group (S‐group), ruderals ecological strategy group (R‐group), and intermediate ecological strategy group (Int‐group; Figure 1 in Han, Huang, et al., 2022). Subsequently, we computed the ecological strategy spectrum for each FDP by dividing the number of species (N) belonging to each ecological strategy group (C‐group, S‐group, Int‐group, and R‐group) by the total number of species (T) in the plot. The ecological strategy spectrum comprises four components: C%, S%, Int%, and R%, which denote the proportions of each ecological strategy group within an FDP. Refer to the Supplementary Materials for a detailed description of the calculation process.

### 2.4 Community functional structure

Community‐weighted mean traits of four functional traits: WD, LPC, LNC and H was used as the descriptors of functional composition.

### 2.5 Statistical analyses

#### 2.5.1 Community‐weighted mean traits

We used “dbFD” function in FD package (Laliberté & Legendre, 2010) to calculate community CWM trait values of WD (CWM‐WD), LPC (CWM‐LPC), LNC (CWM‐LNC) and H (CWM‐H) for each FDP based on abundance.

#### 2.5.2 Effects of functional composition on ecological strategy spectra

To explore and visualize the effect of each CWM trait on the ecological strategy spectra of forests, firstly, we used non‐metric multidimensional scaling (NMDS) ordination using the Bray–Curtis dissimilarity index to group our survey plots based on the ecological strategy spectra of each FDP. Secondly, we model nonlinear correlations in ordination space between the environmental values (i.e., CWM‐WD, CWM‐LPC, CWM‐LNC and CWM‐H) and ecological strategy spectra of forests. NMDS was carried out by the “metaMDS” function in the vegan package (Oksanen et al., 2022). The ecological strategy spectra significance across forest vegetation types was assessed through permutational multivariate analysis of variance (PERMANOVA) via the “adonis” functions in the vegan package. Nonlinear correlations were modeled by the function “ordisurf” in the vegan package. “ordisurf” used a thin‐splined generalized additive model (GAM) to fit a smooth surface for a given CWM trait onto an ordination diagram for detecting distribution trends of species' ecological strategies in forests across different climatic zones.

#### 2.5.3 The relatively importance of CWM traits for variability in ecological strategy spectra

We used “rdacca.hp” function in rdacca.hp package (Lai et al., 2022) to quantify the relatively importance of each CWM trait and group of CWM traits on the ecological strategy spectra of forests, based on datasets of ecological strategy spectra and CWMs for FDPs. Then, we used “upset_vp” function in UpSetVP package (Liu, 2022) to visualize the relative importance of different CWM traits for the ecological strategy spectra of forests.

A Spearman's correlation analysis was performed to reveal the relationships between the CWM traits (i.e., CWM‐WD, CWM‐LPC, CWM‐LNC and CWM‐H) and the ecological strategy spectra of forests (i.e., C%, S%, Int% and R% for FDPs). All statistical analyses were performed using the R environment (R Core Team, 2021) at a significance level of *α* = 0.05.

## 3 RESULTS

### 3.1 Effects of CWMtraits on ecological strategy spectra of forests

The NMDS analysis revealed distinct differences in the ecological strategy spectra among the four forest vegetation types across the four climatic zones (Stress = 0.019), particularly noticeable between Cold‐temperate coniferous forest (CF) and Tropical rainforest (TF), Subtropical evergreen‐deciduous broadleaved mixed forest (SF) and Warm‐temperate coniferous‐broadleaved mixed forest (WF; Figure 1). Species in CF was dominated by stress‐tolerant ecological strategies (i.e., species in the S‐group [S species]) while TF was dominated by species exhibiting competitive ecological strategies (i.e., species in the C‐group [C species]). Both SF and WF predominantly hosted species with intermediate ecological strategies (i.e., species in the Int‐group [Int species]). However, species with ruderal ecological strategies (i.e., species in the R‐group [R species]) differed from the other three ecological strategy groups and were scarce, observed only in SF (see Figure S1).

Nonlinear correlations showed significant associations between the differences in the ecological strategy spectrum among the four forest vegetation types and the CWM traits of wood density (WD), leaf phosphorus concentration (LPC), leaf nitrogen concentration (LNC), and maximum plant height (H) (see Figure 1). Specifically, TF, SF and WF distinctly clustered with low CWM‐WD, while CF clustered with high CWM‐WD (Figure 1a). TF was associated with low CWM‐LPC, whereas CF was linked to high CWM‐LPC. In contrast to TF and CF, SF and WF exhibited intermediate CWM‐LPC (see Figure 1b). CF was characterized by intermediate CWM‐LPC (see Figure 1c).

### 3.2 Relative importance of CWM traits for variability in ecological strategy spectra of forests

**FIGURE 2:**
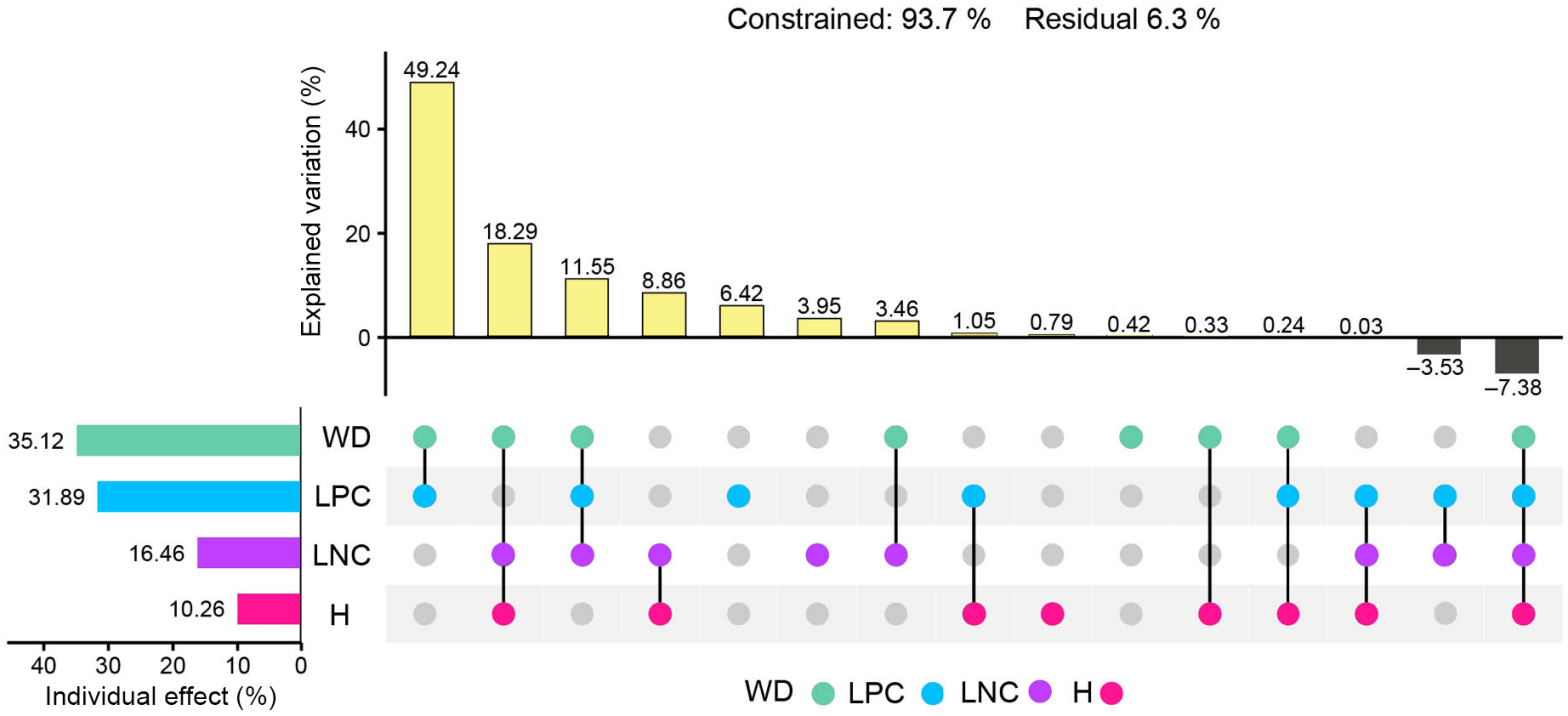
Variance partitioning for representing the relative importance of CWM traits on the ecological strategy spectra of forests. Dot‐matrix plot: each row corresponds to a CWM trait; the isolated dot represents the marginal effect of each CWM trait, lines connecting multiple dots represent the common effect among these corresponding CWM traits. Column diagram on the top: Explained Variation indicates the effect of these corresponding CWM traits. Column diagram on the left: Individual Effect indicates single effect of each CWM trait. Residuals represent the percentage unexplained by these CWM traits. WD, LPC, LNC and H with different colors represented the different CWM traits: WD, aquamarine, wood density; LPC, deepskyblue, leaf phosphorus concentration; LNC, darkorchid, leaf nitrogen concentration; H, deeppink, maximum plant height.

The findings indicated that the four CWMs collectively accounted for 93.7% of the total variation in the ecological strategy spectra of forests, as illustrated in Figure 2. Specifically, the combined influence of CWM‐WD and CWM‐LPC elucidated 49.24% of the observed variation in ecological strategy spectra. Notably, CWM‐WD individually explained up to 35.12% of the variation in ecological strategy spectra. Subsequently, CWM‐LPC and CWM‐LNC emerged as the second and third most influential CWM traits following CWM‐WD, contributing to 31.89% and 16.46% of the variation in ecological strategy spectra, respectively. In contrast, CWM‐H played a minor role, contributing only 10.26% to the observed variation (see Figure 2).

### 3.3 Relationship between CWM traits and ecological strategy spectra of forests

The CWM‐WD, CWM‐LPC, CWM‐LNC and CWM‐H exhibited significant associations with Competitive (C), Stress‐tolerant (S), and Intermediate (Int) ecological strategy groups, as indicated in Table 1. Specifically, CWM‐WD and CWM‐LPC demonstrated significant negative correlations with C and Int‐groups, while showing significant positive correlations with the S‐group. Notably, CWM‐WD exhibited the strongest association with the Int‐group (−0.805) followed by the S‐group (0.774), whereas CWM‐LPC showed the most pronounced association with the C‐group (−0.834) followed by the S‐group (0.451). On the other hand, CWM‐LNC exhibited significant negative correlations with C and S‐groups, along with a significant positive correlation with the Int‐group. In contrast, CWM‐H displayed significant positive correlations with C and S‐groups but was significantly negatively correlated with the Int‐group. Both CWM‐LNC and CWM‐H showed the greatest associations with the Int‐group (0.669 and − 0.620) followed by the S‐group (−0.556 and 0.496; see Table 1).

**TABLE 1:** Spearman's correlation coefficients among community‐weighted mean (CWM) traits and C%, S%, Int% and R% values.

CWM traits	Ecological strategy (%)	Correlation coefficient
WD	C	−0.395***
	S	0.774***
	Int	−0.805***
	R	−0.119^ns^
LPC	C	−0.834***
	S	0.451***
	Int	−0.36***
	R	−0.028^ns^
LNC	C	−0.179*
	S	−0.556***
	Int	0.669***
	R	0.056^ns^
H	C	0.255***
	S	0.496***
	Int	−0.62***
	R	−0.089^ns^

*Note*: *** Indicated significant correlation (*p* < .001), * Indicated significant correlation (*p* < .05), ns indicated non‐significant correlation (*p* > .05).

Abbreviations: H, maximum plant height; LNC, leaf nitrogen concentration; LPC, leaf phosphorus concentration; WD, wood density.

## 4 DISCUSSION

Our main objective was to investigate whether CWM traits could effectively reflect the optimal ecological strategies of species. Utilizing the CSR theory, we demonstrated a robust connection between CWM traits and the ecological strategy spectra of forests. Interestingly, contrary to our second hypothesis, the results indicated that CWM‐WD better reflects species' ecological strategy compared to CWM‐LPC, CWM‐LNC, and CWM‐H. Moreover, in comparison to a single CWM trait, the combined effect of CWM‐WD and CWM‐LPC has a more pronounced impact on the ecological strategy spectra of forests. Further analysis unveiled a strong positive association of both CWM‐WD and CWM‐LPC with the Stress‐tolerant (S) group, coupled with a negative correlation with the Intermediate (Int) group. This suggests that communities with higher CWM‐WD and CWM‐LPC may tend to harbor more S‐selected species than Int‐selected species.

Consistent with our first hypothesis, the results indicate that CWM traits significantly affect the ecological strategy spectra of forests (Figure 1). This suggests that CWM traits have the capacity to elucidate variations in optimal ecological strategies in response to environmental changes. Prior researches (Kandlikar et al., 2022; Muscarella & Uriarte, 2016) support the notion that CWM traits serve as effective descriptors, reflecting the adaptive strategies of species. According to the mass ratio hypothesis (Grime, 1998), ecosystem processes in plant communities are assumed to be dictated by the traits of the most abundant species, those with specific resource use strategies. In other words, CWM traits can indicate the dominant ecological strategy of species in plant communities. Likewise, the ‘CWM‐optimality’ hypothesis (Muscarella & Uriarte, 2016) also partly confirms that CWM traits in a community can reflect the optimal strategies for a given environment. Our findings align with these previous studies, suggesting that understanding the relationships between CWM traits and ecological strategies can facilitate the prediction of species assembling in specific communities.

One of the most significant findings in our study is that CWM‐WD (35.12%), rather than CWM‐H (10.26%), stands out as the most crucial individual CWM trait in explaining the variation in the ecological strategy spectra of forests (Figure 2). CWM‐WD was a more effective indicator for optimal ecological strategies in forest communities at regional scales. This finding diverges from our second hypothesis but aligns with previous research that identifies wood density as a reliable predictor for fundamental plant characteristics, including resistance to xylem damage and growth rate (Preston et al., 2006). Moreover, studies by Diaz et al. (2016) have highlighted wood density as a vital indicator of construction costs and structural strength, positively linked to plant mechanical strength, hydraulic safety, and resistance to biotic agents among woody species (Chave et al., 2009; Zanne et al., 2010). Although plant height is closely related to water use efficiency and light competition ability (Falster et al., 2017; Klein et al., 2015), wood density also possesses these functions and has additional functionalities (Chave et al., 2009). Additionally, compared to H, there are stronger correlations between WD and functional traits that determine ecological strategies (i.e., LA and LDMC; Figure S2). In light of these considerations, we propose that regarding CWM‐WD as a key indicator reflecting the assembly of species' ecological strategies is both rational and acceptable for woody species in forest communities across four climatic zones.

Further analysis revealed a strong correlation between CWM‐WD and the S and Int‐groups (Table 1). Our results directly indicated that forest communities with higher CWM‐WD were generally dominated by species in the S‐group. Several factors contribute to this pattern. Firstly, WD exhibits a positive association with LDMC (Figure S2), and stress‐tolerant species are closely linked to LDMC (Pierce et al., 2013). Secondly, this observation aligns with the “mass ratio hypothesis” (Grime, 1998), suggesting that higher CWM‐WD in forest communities corresponds to the presence of more plants with high wood density. Previous research has indicated that plants with higher wood density confer low growth rates and stress resistance (Hacke & Sperry, 2001; Poorter et al., 2017; Sperry et al., 2008). S‐selected species are characterized by slow growth and high stress tolerance in challenging environments (Pierce et al., 2017). Therefore, plant communities with higher CWM‐WD tend to have more S‐selected species. Contrary to the S‐group, CWM‐WD was negatively correlated with the Int‐group. This can be attributed to the intermediate characteristics of the Int‐group, which falls between the extremes of the true ecological strategy groups (C, S, and R). Species in the Int‐group exhibit intermediate responses, such as wood density intermediate values, to competition, disturbance, and stress of similar intensities (Grime, 2001; Han, Huang, et al., 2022). Consequently, forest communities with a significant number of species in the Int‐group may exhibit lower CWM‐WD. Furthermore, our previous studies have consistently demonstrated that a substantial proportion of woody species in these four forest types belongs to the S and Int‐groups (Han, Huang, et al., 2022). This finding partly contributed to the substantial impact of CWM‐WD on the variation in the ecological strategy spectra of forests.

The combined effects of CWM‐WD and CWM‐LPC have the greatest impact on the ecological strategies of forest communities (49.24%; Figure 2). Plant traits associated with the stem and leaf economics spectrum are functional and significantly influence plant ecological strategies (Baraloto et al., 2010; Wright et al., 2004). The examination of “the wood economics spectrum” (Chave et al., 2009) encompasses wood properties related to water transport, storage, mechanical strength, and defense mechanisms, forming a triangle (see fig. 4 in Chave et al., 2009) that symbolizes the role of wood in crucial plant ecological functions‐competitive ability, stress resistance, and disturbance resilience. Intriguingly, this happens to correspond to CSR theory (competitor, stress‐tolerant, and ruderal; Grime, 1974). Additionally, wood density, serving as an integrator of wood properties and stem functional traits, has been validated as one of the most reliable indicators of growth rate and mortality (Poorter et al., 2008) and is instrumental in reflecting plant performance (Chave et al., 2009). On the other hand, robust associations have been identified between leaf traits and life‐history strategies through multivariate approaches in tropical forests with similar environmental conditions (Rüger et al., 2018). Leaf phosphorus concentration, as a key indicator representing the leaf economics spectrum, plays a pivotal role in plant adaptation strategies to environmental conditions (Pan et al., 2023; Wright et al., 2004). Our findings reinforce these conclusions and underscore the importance of wood density and leaf phosphorus concentration in comprehending the plant community response to ongoing global changes on a large scale (Chave et al., 2009).

Overall, our study delves into the reciprocal relationship between ecological strategy and community functional structure in regional forest communities across four climatic zones. The results illuminated tight associations between CWM traits and the ecological strategy spectra of forests. Comparatively, considering both CWM traits of stem and leaf traits collectively offers a potentially more accurate predictive capacity for changes in ecosystem properties and functions within forest communities than focusing on individual stem or leaf CWM trait. Moreover, our findings validate CWM‐WD as a more effective indicator for optimal ecological strategies in forest communities at regional scales. This insight stands to aid ecologists in advancing their comprehension of species adaptation and community assembly within forest ecosystems. However, certain limitations persist in this study. Primarily, the analysis exclusively concentrates on aboveground functional traits, omitting belowground functional traits such as root traits (e.g., specific root length). Addressing this gap is a focal point for our future research endeavors. On the other hand, these abovementioned outcomes may be partially attributed to species' growth forms (Baraloto et al., 2010; Diaz et al., 2016) and the scale of the study (Westoby et al., 2002). In essence, while our study underscores the effectiveness of CWM‐WD in explaining variation in the ecological strategy of forest communities, divergent growth forms (e.g., understory herbaceous plants), study scales, or both, could yield disparate results. Consequently, further research with larger sample sizes may be necessary to provide a more comprehensive interpretation of these findings in the future.

## AUTHOR CONTRIBUTIONS

**Xin Han:** Formal analysis (lead); investigation (equal); methodology (equal); writing – original draft (lead); writing – review and editing (equal). **Jie Yao:** Data curation (equal); funding acquisition (lead); investigation (equal); writing – review and editing (equal). **Ruixue Wang:** Formal analysis (equal); writing – original draft (equal); writing – review and editing (equal). **Yue Xu:** Data curation (equal); formal analysis (equal); investigation (equal); visualization (supporting); writing – review and editing (supporting). **Jihong Huang:** Data curation (equal); formal analysis (equal); investigation (equal); visualization (equal); writing – review and editing (supporting). **Yi Ding:** Data curation (equal); formal analysis (equal); investigation (equal); visualization (equal); writing – review and editing (supporting). **Runguo Zang:** Conceptualization (lead); supervision (lead); writing – review and editing (lead).

## CONFLICT OF INTEREST STATEMENT

The authors declare that they have no conflict of interest.

## Supporting information


Data S1.

## Data Availability

All data that support the finding of this study can be available from the following https://doi.org/10.5061/dryad.qfttdz0js.
