# Supplementary material for: Effects of functional composition on plant competitors, stress‐tolerators, ruderals ecological strategies in forest communities across different climatic zones
Source: Ecol Evol. 2024 Sep 3;14(9):e11580. doi: 10.1002/ece3.11580 (PMC11371659; doi:10.1002/ece3.11580)
Supplement: Supplementary file 1 — Data S1. [file ECE3-14-e11580-s001.pdf]

The formulas and explanations for the calculations of ecological strategy spectrum:

$$\text{C\%} = \frac{N_C}{T}$$

$$\text{S\%} = \frac{N_S}{T}$$

$$\text{Int\%} = \frac{N_{Int}}{T}$$

$$\text{R\%} = \frac{N_R}{T}$$

Where:

C%, *S%*, *Int%* and *R%* represent the proportions of the C-group, S-group, Int-group, and R-group species respectively, within an FDP. N_C, N_S, N_{Int}, and N_R denote the number of species in the C-group, S-group, Int-group, and R-group respectively. *T* is the total number of species in the plot.

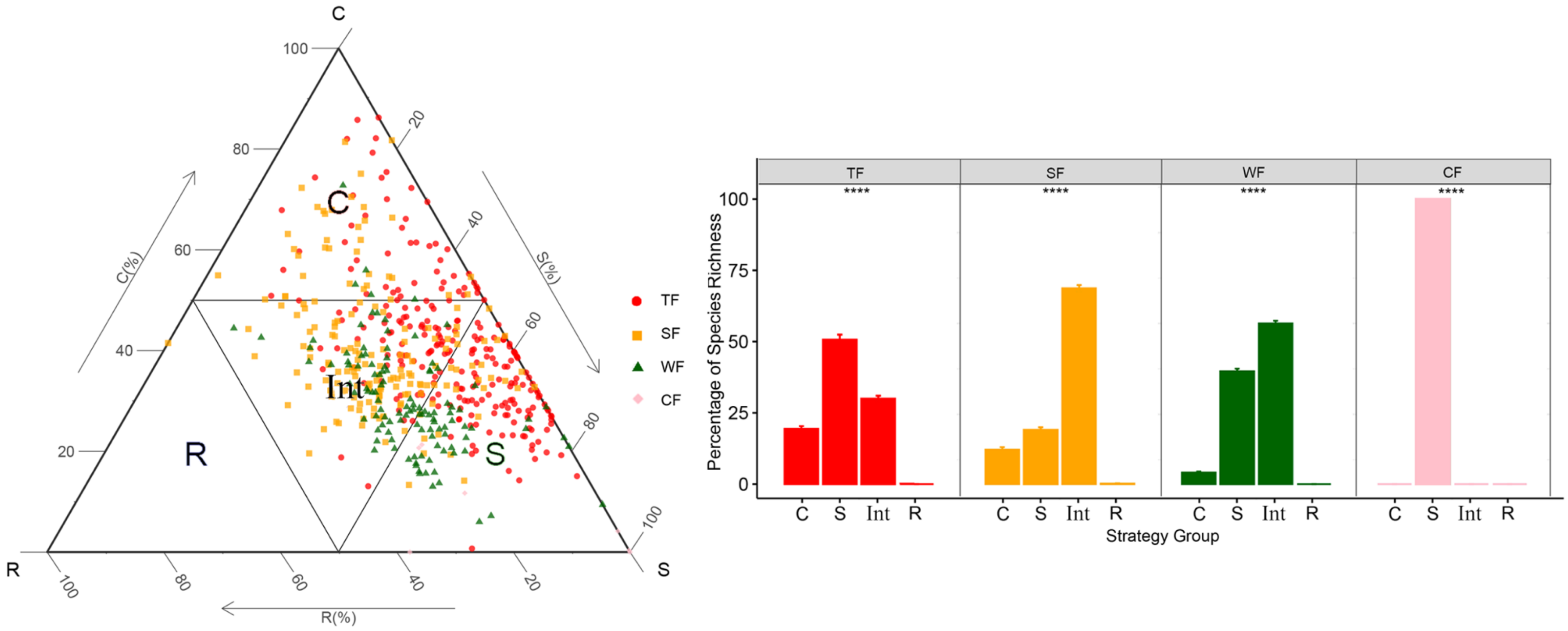

Fig. S1 Left: The distribution of all species and the delimitation of the four ecological strategy groups in the CSR triangle. Right: Bar plots for showing species' percentage of each ecological strategy group of ecological strategy spectrum of four forest types. ****$p < 0.001$ means significant difference among ecological strategy groups. Different colors and symbols indicate species in different forest types: TF, red, circles, tropical forest; SF, yellow, squares, subtropical forest; WF, dark green, triangles, warm-temperate forest; CF, pink, diamonds, cold-temperate forest. C, S, Int and R indicated different ecological strategy groups: C, competitive ecological strategy group; S, stress-tolerant ecological strategy group; Int, intermediate ecological strategy group; R, ruderals ecological strategy group.

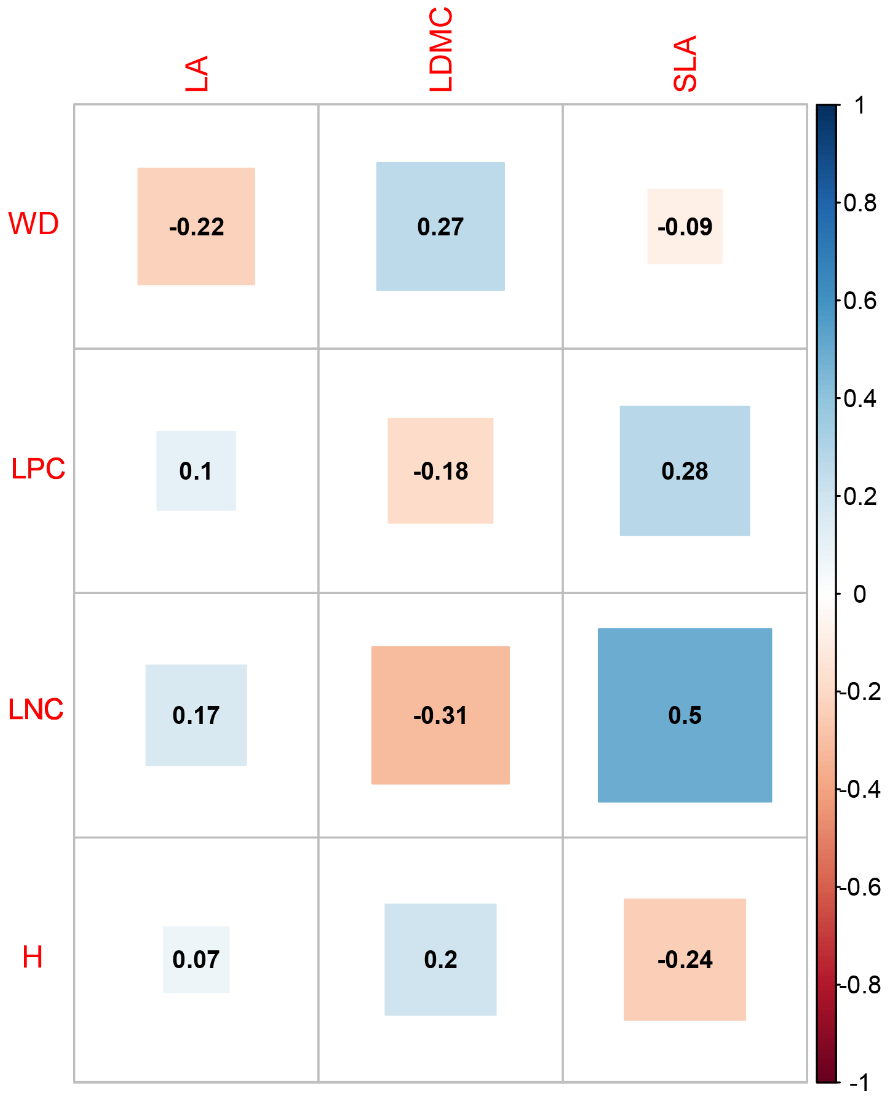

Fig. S2 The relationships between explaining functional traits (WD, LPC, LNC, and H) and functional traits (LA, LDMC and SLA) determined ecological strategies. WD, wood density; LPC, leaf phosphorus concentration; LNC, leaf nitrogen concentration; H, maximum plant height; LA, leaf area; LDMC, leaf dry mass content; SLA, specific leaf area.
